# Effect of a behavior change and hardware intervention on safe child feces management practices in rural Odisha, India: a cluster-randomized controlled trial

**DOI:** 10.1186/s12889-024-19272-5

**Published:** 2024-08-27

**Authors:** Gloria D. Sclar, Valerie Bauza, Alokananda Bisoyi, Fiona Majorin, Hans-Joachim Mosler, Thomas F. Clasen

**Affiliations:** 1 Gangarosa Department of Environmental Health, Rollins School of Public Health, Emory University, Atlanta, GA USA; 2 Department of Psychology, University of Zürich, Zürich, Switzerland; 3 Independent Consultant, Berhampur, Odisha India; 4 Department of Disease Control, Faculty of Infectious and Tropical Diseases, London School of Hygiene and Tropical Medicine, London, UK; 5 RanasMosler, Bergheimstrasse 8, Zürich, 8032 Switzerland

**Keywords:** Behavior change, Child health, India, Safe disposal, Sanitation, Toilet training

## Abstract

**Background:**

Poor child feces management contributes to enteropathogen exposure and, consequently, is associated with diarrheal disease and negative impacts on child growth. Despite high latrine coverage, only 37% of Indian households safely dispose of their child’s feces into a latrine or have the child use the latrine, with the lowest rate in the state of Odisha at 12%. We evaluated a behavior change and hardware intervention designed to improve caregiver safe disposal of child feces and child latrine use.

**Methods:**

We conducted a cluster-randomized controlled trial among 74 villages in rural Odisha, India. Eligible villages previously participated in a water and sanitation infrastructure program. Following a baseline survey, half the villages were assigned to intervention and half to control. Caregivers of children < 5 years old from households with a latrine were eligible to participate. The intervention included five behavior change activities. Hardware was provided at the first activity, based on child age, to aid safe disposal and latrine training (wash basin and bucket with lid for children < 7 months old; latrine training mat platform with removable tray for children 7 to 48 months old). The primary outcome was caregiver reported ‘safe disposal’ as defined by the WHO/UNICEF Joint Monitoring Programme (JMP) which encompasses two behaviors: caregiver disposal of child’s feces into a latrine and child latrine use. Safe disposal was measured four to six months after intervention delivery (endline).

**Results:**

Endline analysis included 665 intervention caregivers (840 children) and 634 control caregivers (785 children). Prevalence of JMP-defined safe disposal was 1.16 times greater in the intervention arm compared to control (77.7% vs. 65.9%; prevalence ratio [PR] 1.16, 95% CI 1.04–1.29), with higher prevalence of caregiver safe disposal (18.6% vs. 13.6%; PR 1.46, 95% CI 1.12–1.92) but no significant difference in child latrine use (59.0% vs. 52.2%; PR 1.06, 95% CI 0.95–1.18). When restricted to children < 3 years old, JMP-defined safe disposal was 1.42 times greater (67.5% vs. 46.7%; PR 1.42 95% CI 1.21–1.67) with higher prevalence of both caregiver safe disposal (34.6% vs. 25.7%; PR 1.44, 95% CI 1.11–1.86) and child latrine use (32.9% vs. 20.9%; PR 1.41, 95% CI 1.08–1.83).

**Conclusions:**

The intervention increased JMP-defined safe disposal, with substantial improvements in both caregiver safe disposal and child latrine use among children < 3 years old. While future research is needed to demonstrate sustainability of these effects, our results suggest a potentially scalable intervention for improving child feces disposal and reducing disease.

**Trial registration:**

This trial was retrospectively registered at ISRCTN15831099 on 18/02/2020, which was approximately two months after the first participant was recruited for the baseline survey on 02/12/2019.

**Supplementary Information:**

The online version contains supplementary material available at 10.1186/s12889-024-19272-5.

## Background

The world has made great gains in sanitation access and reducing adult open defecation, but the health risk posed by child feces is often ignored [1]. In many settings, children under five defecate around the household and their feces are unsafely disposed in a nearby open area or water source [2]. Research shows child feces are a more common source of fecal contamination in the household environment than adult feces [3]. This fecal exposure poses a health risk to all household members, but especially to young children who interact with their surroundings through mouthing and other exploratory behaviors [4, 5]. Consequently, the unsafe disposal of child feces is associated with diarrheal disease and negative impacts on child growth, especially stunting [2, 6]. To mitigate this risk, caregivers must safely dispose of their child’s feces into a latrine or children learn to use the latrine.

Although India has seen substantial increases in sanitation coverage, safe child feces disposal and child latrine use remain particularly low. In the 2019–2021 National Family Health Survey, 82.5% of Indian households had access to a latrine [7]. Despite this, among households with a child under five, only 18.7% safely disposed of their child’s feces into a latrine and only 18.0% reported their child used the latrine. The state of Odisha had the lowest rate with 5.3% safe child feces disposal and 6.5% child latrine use [7]. The practice of burying child feces is rare in India with only 1.6% of households reporting this practice (1.5% in Odisha) [6].

Child feces in rural India are unsafely managed even when households have improved water and sanitation facilities. An evaluation was conducted in rural Odisha of a community-based intervention, known as Movement and Action Network for Transformation of Rural Areas (MANTRA), wherein households gained an improved latrine with attached bathing room and piped water supply. Among children under five in intervention households only 6.0% had their feces safely disposed and 34.8% used the latrine [8]. The Indian, Canadian, and American Academies of Pediatrics all note that children are typically developmentally ready to start toilet training around age two [9–11]. However, even among 2 and 3-year-olds, latrine use was low (33.7% and 57.2%, respectively) indicating a lack or delay of latrine training [8]. This suggests infrastructure is not the only barrier to performing these behaviors and interventions must employ behavior change techniques that address underlying psychosocial factors in the mindset of caregivers.

Aside from the method of disposal, many other child feces management (CFM) practices can also contaminate the household environment. How a caregiver manages her child’s feces consists of a string of behaviors (i.e. CFM practices) with multiple points of potential exposure: where and on what child defecates, how feces are handled, where feces are disposed, if and how tools are used and cleaned, and more [12, 13]. A cross-sectional study in rural Odisha measured fecal contamination from different CFM practices. Child defecation on finished floor or ground, even when paper was laid down beforehand, increased *E. coli* contamination of the surface and use of unsafe tools to pick up the feces — paper, plastic bag, straw/hay — increased *E. coli* contamination of the caregiver’s hands [14]. There is a need for interventions that consider more than just where feces are disposed but promote fully safe CFM practices.

We partnered with the Odisha-based NGO, Gram Vikas — that developed and delivers the MANTRA program — to design a behavior change and hardware intervention that promoted safe CFM practices, with an emphasis on caregiver safe disposal and child latrine training at an earlier age. The intervention was specifically developed for households that already had improved water and sanitation facilities. The aim of this study was to evaluate the effect of the intervention on prevalence of safe CFM behaviors and to examine the psychological mechanism by which the intervention may have led to behavioral change.

## Methods

Details of the study design, setting, intervention, and corresponding process evaluation are reported in Sclar, Bauza [15]. We summarize these briefly below.

### Study design and participants

The study was designed as a cluster-randomized controlled trial among 74 villages in rural Odisha, India. Eligible villages (i.e. clusters) were randomly selected from a list of 501 villages in Ganjam and Gajapati districts that Gram Vikas had previously engaged in the MANTRA program. To mitigate spillover, we also required all villages to have their own Anganwadi center (government-run daycare and preschool center); if multiple villages shared an Anganwadi center it could cause spread of intervention messaging. The study took place between December 2019 to September 2021. We conducted a household census in each village at baseline and endline to identify all eligible caregivers. At baseline, we enrolled caregivers of children < 5 years from households with a latrine and did the same at endline but restricted to caregivers of children who were < 5 years *at the time of intervention delivery*. Since endline data collection took place four to six months after intervention delivery, children could be up to 5 years and 6 months at endline. Participation at endline was not conditioned on enrollment at baseline. For the latrine criteria, in the majority of cases the caregiver’s household owned the latrine; however, there were instances where the latrine was owned by another household, typically a family member, but the caregiver’s household used the latrine for their daily needs. The effect of the intervention was measured by comparing intervention and control participants at endline only. This trial was retrospectively registered on 18/02/2020 at ISRCTN15831099.

### Randomization and masking

After baseline data collection, villages were randomly assigned by author GDS on a 1:1 basis to either intervention or control arm using a computer-based random number generator. To help ensure balance on a variety of external factors, randomization was stratified by geographic-demographic (‘geo-demo’) group. Villages were categorized into one of five distinct geo-demo groups which shared characteristics related to geography, religion and caste demographics, village size, and market access. It was not possible to mask participants to village assignment due to the nature of the intervention. The enumerator team was different from the intervention delivery team.

### CFM intervention and delivery

We collaborated with Gram Vikas to design the intervention and applied the Risks, Attitudes, Norms, Ability, Self-regulation (RANAS) approach to behavior change [16]. We focused on two specific behaviors — caregiver safe disposal of child feces into the latrine and child latrine training. As detailed in Sclar, Bauza [15], the intervention was developed based on extensive formative research and piloting. Along with qualitative research, we used the baseline survey data to examine which psychosocial factors are associated with safe disposal and child latrine training in order to select the right behavior change techniques (BCTs) for the intervention [17]. The five intervention activities and their BCTs are detailed in Table 1. The intervention was delivered to primary caregivers of children < 5 years old, typically mothers, with engagement of other caregivers such as fathers and grandmothers in certain activities to foster social support around CFM. For household-level activities, the behavioral messaging was tailored to which behavior the caregiver was currently focused on. In all activities, the behavioral messaging also encouraged caregivers to adopt a fully safe CFM practice and discussed *transitions* in CFM as the child grows and develops (i.e. when to start latrine training).

**Table 1:** Intervention matrix describing each activity, the BCTs employed, and psychosocial factors targeted

Activity name (communication channel)	Engaged participants	BCTs*	Activity description	RANAS psychosocial factors
**Hardware and Action Knowledge** **(Opening Meeting)**	Caregivers of children under 5	*Part A: Video and discussion* • *BCT 3: Inform about risk* • *BCT 5: Inform about costs & benefits* • *BCT 8: Describe feelings about performing behavior* • *BCT 13: Provide positive group identity* *Part B: Hardware demo and practice* • *BCT 15&16: Provide infrastructure & instruction* • *BCT 18: Prompt guided practice* *Part C: Group commitment* • *BCT 10: Prompt public commitment*	Part A: Discuss and watch video story of two families – one family safely manages their child’s feces and experiences many benefits while the other family does not and faces consequences. Part B: Mobilizer uses banner with illustrations to explain how to use each hardware; participants then demonstrate the practice and receive feedback and praise; hardware is distributed to caregivers. Part C: Caregivers make commitment in front of each other that they will practice safe disposal and/or latrine training (so that all child feces end up in latrine).	✓ Vulnerability ✓ Beliefs about costs & benefits ✓ Feelings ✓ Personal importance ✓ How-to-do knowledge ✓ Confidence in performance ✓ Others’ behavior
**Building Self-Efficacy and Goal Setting** **(Household Visits x2)**	Families of children under 5	*Part A: Behavior reflection* • *BCT 28: Feedback on performance* *Part B: Discuss challenges* • *BCT 30: Prompt coping with barriers* • *BCT 24: Reattribute past successes & failure* *Part C: Set behavioral goal* • *BCT 35: Prompt goal setting* *Part D: Family support* • *BCT 11: Inform about others’ approval* • *BCT 21: Organize social support*	Part A: Caregiver demonstrates her CFM practice (with hardware); mobilizer provides feedback as needed and praise. Part B: Discuss challenges caregiver is facing with her practice. For safe disposal, create a “barrier plan” for the biggest challenges. For latrine training, explain setbacks are normal and reflect on a successful training moment. Part C: Create “goal tracker” using two empty containers and stones. Caregiver sets goal to safely dispose/latrine train every day until mobilizer’s next visit. (In visit #2, reflect on goal and potential need to transition CFM practice as child grows). Part D: Mobilizer explains CFM is a family responsibility. Encourages other family members (e.g. father, grandmother) to discuss how they will support the primary caregiver.	✓ Action control ✓ Barrier planning ✓ Confidence in continuation ✓ Commitment ✓ Others’ approval ✓ Confidence in performance
**Caregiver Support** **(Group Meeting)**	Caregivers of children under 5	• *BCT 27: Prompt self-monitoring of behavior* • *BCT 21: Organize social support*	Caregivers reflect on how well they followed their goal tracker. Mobilizer then facilitates group discussion on challenges with safe disposal/latrine training and caregivers share strategies and supportive words. Close meeting with discussion about how to transition to a new CFM practice as your child grows (i.e. when to begin latrine training).	✓ Action control ✓ Confidence in performance
**Celebrating ‘Safe CFM Families’** **(Closing Meeting)**	Families of children under 5	• *BCT 13: Provide positive group identity* • *BCT 11: Inform about others’ approval*	Mobilizer facilitates meeting where caregivers and their family members share positive reflections on adopting safe disposal/latrine training. Prominent village stakeholders (e.g. Ward member, Anganwadi teacher, ASHA worker) provide families with a celebratory certificate.	✓ Personal importance ✓ Others’ approval

BCT = behavior change technique; *The BCTs come from the RANAS practical guide [12]

The intervention included provision of hardware — a BCT that addresses the ‘ability’ behavioral factor — in order to aid caregivers with their new practices. Hardware was designed and selected through a co-design process with caregivers, including user-centered design (UCD) sessions and piloting [18, 19]. Caregivers of babies (0 to < 7 months) were provided a bucket with lid to safely store soiled cloth and a basin to wash cloth and safely dispose of the wash water into the latrine. Caregivers of children (7 to < 48 months) were provided a latrine training mat platform with removable tray, which was designed to “grow” with the child as it could be used in two ways: [1] placed over the ground with the tray underneath to aid safe disposal or [2] placed over the latrine squat plate without the tray to aid child latrine training. Hardware descriptions, photos, and use instructions are provided in Figure S1.

The Gram Vikas implementing team consisted of nine mobilizers (six women and three men) and two managers who provided oversight and support. Mobilizers underwent a week-long training where they reviewed the “implementer’s guide” for each activity and practiced via role-play. Each mobilizer was assigned three to five intervention villages and delivered the CFM intervention to all present caregivers with a child < 5 years old (approximately 40 to 120 caregivers per mobilizer). The intervention took place between December 2020 to April 2021. No activities took place in the control villages. Mobilizers took on average one month to deliver each activity across their assigned intervention villages, thus caregivers had monthly mobilizer contact. The implementing team also met monthly to reflect on successes and challenges.

### Data collection

Data was collected by a team of trained enumerators fluent in the local languages and supervised by author AB. Surveys were developed in English, translated into Odia and administered using the mobile application Open Data Kit (ODK) Collect. Baseline took place between November 2019 to February 2020 and endline between July to September 2021. Enumerators went door-to-door and assessed every household for eligibility. Confirmed eligible households were asked to participate. The target respondent was the primary caregiver of the child but if unavailable then a secondary caregiver was asked to participate. Upon verbal consent, the caregiver answered questions on demographics, household water and sanitation infrastructure, CFM practices, social support with CFM, and psychosocial factors related to CFM. After the survey, the enumerator completed a structured spot-check of the household’s latrine, bathing room, and compound. At endline, intervention caregivers were also asked about attendance at activities (after CFM practices section).

### Primary outcomes

The primary outcome was safe disposal of child feces for children < 5 years old as defined by the WHO-UNICEF Joint Monitoring Program on Water, Sanitation and Hygiene (JMP) [20]. Two distinct behaviors are considered safe disposal by the JMP: (a) *caregiver safe disposal* of child feces into a latrine and (b) *child latrine use*. These two behaviors (‘caregiver safe disposal’ and ‘child latrine use’), together with the primary endpoint of either (a) or (b) to match the JMP definition of ‘safe disposal,’ were analyzed as binary outcomes based on caregiver self-report. In the trial surveys the caregiver was first asked, “The last time [name of child] defecated, where did [name of child] defecate?” If the caregiver did not report “in latrine” then a follow-up was asked, “Where was [name of child]‘s feces disposed?” Caregivers reported on the CFM practice for each child < 5 years old who resided in the household. We analyzed children < 3 years old separately since they had the greatest room for improvement with regard to both behaviors.

### Secondary outcomes

We measured several secondary outcomes to examine broader intervention impacts. Caregivers reported on their full CFM practices and we assessed behaviors along this “exposure pathway”: (1) where and on what child defecates, (2) how feces are handled, (3) where feces are disposed, (4) if and how tools are used and cleaned, (5) how and where anal cleansing takes place, and (6) whether caregivers and children wash their hands afterwards. We also measured caregiver’s received social support with CFM since the intervention was intended to bolster support. As a possible unintended consequence of the intervention, we assessed caregiver’s perceived workload of their CFM practice. As a potential downstream effect of the intervention, household fecal contamination was measured by enumerators conducting a spot-check of the household compound and recording any human or animal feces observed.

Lastly, we measured psychosocial factors which can be viewed as the intermediate outcomes of the CFM intervention whereby a change in the mindset of caregivers must first take place before behavioral change. There were two different psychosocial questionnaires —one on child feces disposal and another on latrine training — but caregivers only answered one. Caregivers who perceived their child too young to learn to use the latrine answered the disposal version. The questionnaires examined the different psychosocial factors outlined in the RANAS approach, were informed by qualitative research, and used 5-point Likert responses.

### Statistical analyses

The sample size was based on prevalence of JMP-defined safe disposal among children < 5 years old. We used formula five from Rutterford, Copas [21] and estimated the baseline prevalence and intra-class correlation (ICC) from prior sanitation trials in Odisha [8, 22]. We assumed a 15 percentage point increase (prevalence ratio [PR] 1.37) as a desirable and reasonable level of behavior change. Assuming nine eligible households per village (i.e. cluster size), ICC of 0.103, 10% loss to follow-up, 40.7% baseline prevalence of JMP-defined safe disposal, 80% power, and p-value of 0.05, we calculated 37 villages per arm. To account for unequal cluster sizes, after baseline we ran simulations that repeatedly resampled the 37 villages per arm and found the median effect size was 16 percentage points (PR of 1.39).

We conducted intention-to-treat (ITT) analyses to calculate prevalence ratios of JMP-defined safe disposal, caregiver safe disposal, and child latrine use between intervention and control children at endline. We used log-binomial models and generalized estimating equations (GEE) with robust standard errors to account for clustering. Models were adjusted for two village-level variables: baseline prevalence and geo-demo group. If a model did not converge, then Poisson regression was used instead of log-binomial. We used the same model specifications for per-protocol analyses to estimate the effect of high intervention adherence — caregivers attending at least three activities and receiving hardware if their child was age eligible.

We also examined effects by child gender and age and on secondary outcomes. The same ITT model specifications were used to calculate prevalence ratios by child gender and prevalence ratios restricted to children < 3 years old. Child age was also examined categorically with age groups aligning with developmental milestones related to CFM (e.g. crawling, walking, squatting) [23]. Cluster-adjusted two-sample tests of proportions or t-tests were used to examine prevalence difference between intervention and control at endline for each child age group and these secondary outcomes: CFM exposure pathway behaviors, caregiver received social support, caregiver perceived CFM workload, and observed feces in the household compound.

We used mediation analysis to understand the mechanism for how the intervention led to behavior change. In this analysis we can identify which psychosocial factors were trigged by the intervention (path a), which were associated with the behavior (path b) and, most importantly, which mediated the intervention effect on the behavior (indirect effect ab). We separately examined caregiver safe disposal consistency (continuous outcome measured by three-point Likert — always safely disposed in the past week, sometimes, never) and child latrine training intensity (continuous outcome measured by six-point Likert referring to how often in the past week the caregiver took their child to the latrine when they needed to defecate and taught them how to use it — never, almost never, seldom, sometimes, often, (almost) always). Analyses were restricted to per-protocol. First, we ran simple mediation analyses to identify psychosocial factors significantly associated with the intervention. Then we ran a parallel mediation analysis with study arm as the independent variable, significant psychosocial factors as mediators, and behavior as the dependent variable. We used bootstrapped confidence intervals to determine significance of mediated effects.

Lastly, as a sensitivity analysis we fitted stratified ITT models to compare differences in intervention effects between caregivers who received both the baseline and endline surveys to those caregivers who received only the endline survey. The WHO declared COVID-19 a pandemic in March 2020, approximately 1 month after the baseline survey completed. In India, the pandemic led to a national lockdown and mass internal migration as migrant workers left the cities and returned to their villages [24]. Those caregivers present for both surveys may have been different from caregivers present only at endline, as the latter may be from migrant families.

The analysis plan is registered with 3ie RIDIE (STUDY-ID-61d2b60449ba1). Analyses were conducted in STATA version 17, except for the mediation analyses which were run in SPSS v26 using the statistical application PROCESS [25].

### Patient and public involvement

Gram Vikas leadership and staff were involved in intervention design and delivery approach. Stakeholder workshops — with representatives from government, community-based development organizations, and international NGOs (e.g. WaterAid, UNICEF) — were held at the start and end of the study to elicit input on intervention design, provide feedback on trial results, and promote dissemination. A researcher reflexivity statement is provided in Supplemental File 2.

## Results

### Trial participants

**Fig. 1:**
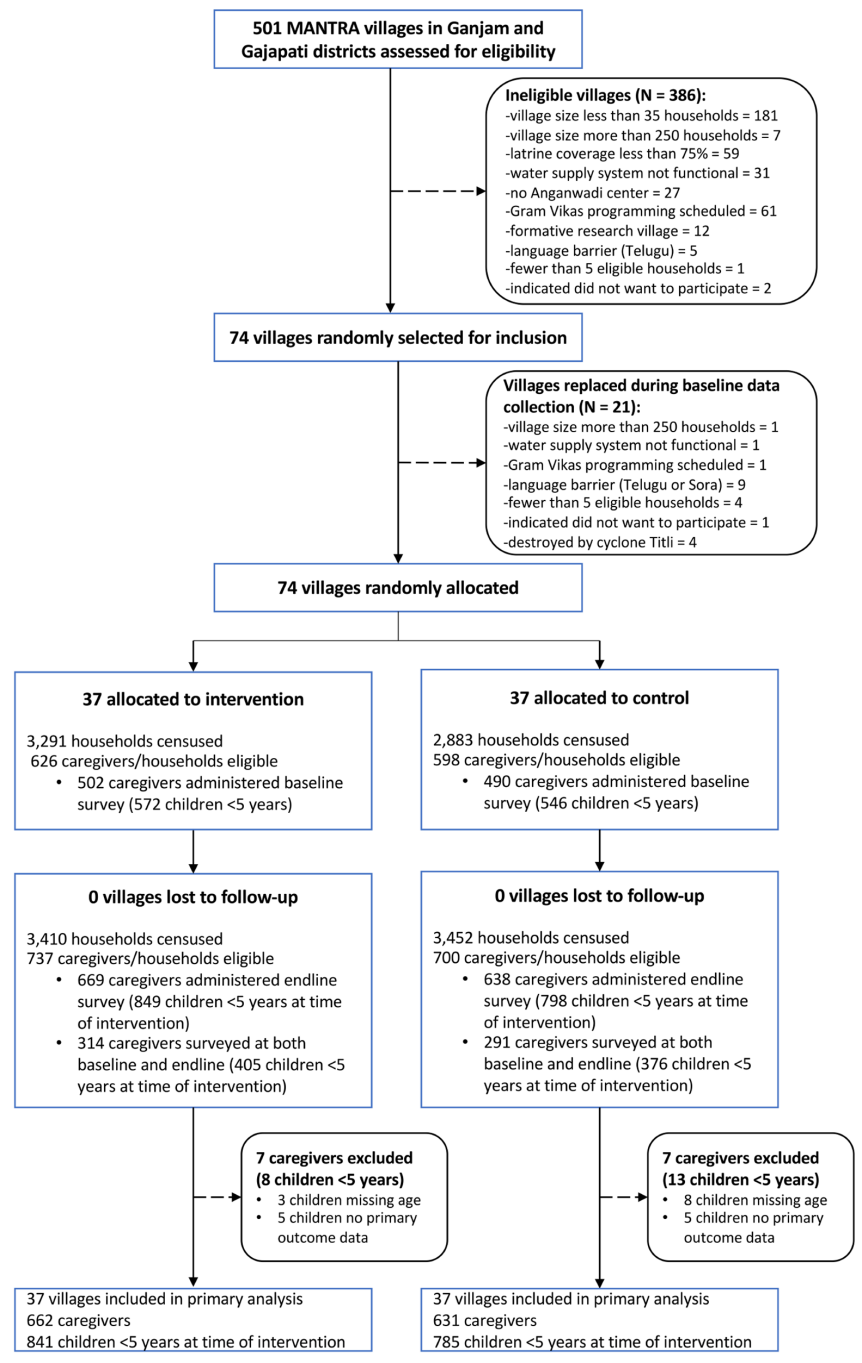
Trial profile

Figure 1 presents the trial flow diagram. At baseline, we enrolled 502 caregivers in the intervention arm and 490 caregivers in the control arm. Baseline demographics and household water and sanitation characteristics were balanced across arms (Table 2). At endline, we enrolled 669 caregivers in the intervention arm and 638 caregivers in the control arm. Four caregivers in each arm were dropped from the analysis because of missing data for child age or the primary outcome JMP-defined safe disposal. This resulted in an analytical sample of 665 intervention caregivers reporting on 840 children and 634 control caregivers reporting on 785 children.

**Table 2:** Baseline characteristics of intervention and control villages, households, caregivers, and children

	Total		Intervention		Control
	**N**		n (%) / mean (SD)		n (%) / mean (SD)
**Village characteristics**					
Village size (total # of households)	74		94.0 (56.0)		91.4 (48.8)
**Household characteristics**					
Household religion	942				
Hindu			387 (79.8%)		365 (79.9%)
Christian			98 (20.2%)		88 (19.3%)
Other			0 (0.0%)		2 (0.4%)
No religion			0 (0.0%)		2 (0.4%)
Household caste^*^	945				
General			107 (22.0%)		75 (16.3%)
Scheduled Caste			28 (5.8%)		51 (11.1%)
Other Backward Caste			182 (37.4%)		141 (30.7%)
Scheduled Tribe			120 (24.7%)		137 (39.8%)
Don’t know/other			48 (9.9%)		55 (12.0%)
Refused			1 (0.2%)		0 (0.0%)
Household wealth (asset index pca score)	944		2.2 (0.9)		2.2 (0.9)
Only one child < 5 years old	972		401 (80.8%)		386 (81.1%)
Household size	944		5.9 (2.21)		5.8 (2.1)
**Caregiver and child characteristics**					
Primary female caregiver	992		446 (88.8%)		422 (86.1%)
Caregiver age	991		28.6 (7.9)		29.0 (8.5)
Caregiver years of schooling	937		6.2 (4.2)		6.2 (4.4)
Caregiver employment	945				
Employed (outside the home)			39 (8.0%)		48 (10.5%)
Employed (inside home)			192 (39.5%)		177 (38.5%)
Unemployed			255 (52.5%)		234 (51.0%)
Caregiver latrine use	940		339 (70.3%)		327 (71.4%)
Child’s age (months)	1117		30.9 (16.6)		30.1 (16.6)
Child female	1118		277 (48.4%)		267 (48.9%)
**WASH characteristics**					
Household has functional piped water	947		430 (88.3%)		409 (88.9%)
Latrine in or within 50 ft of household	932		423 (88.1%)		383 (84.7%)
Latrine has two pits	911		316 (67.4%)		284 (64.3%)
Latrine structure fully intact	909		383 (82.9%)		359 (80.3%)
Latrine has tap inside	920		401 (85.3%)		394 (87.6%)
Latrine has water (tap tested)	920		262 (55.7%)		214 (47.6%)
**Outcomes**					
JMP-defined safe disposal	1104		294 (52.3%)		268 (49.5%)
Caregiver safe disposal	1104		57 (10.1%)		49 (9.0%)
Child latrine use	1109		237 (42.0%)		219 (40.3%)

^*^We used the Government of India’s classification system for caste groups

### Intervention fidelity

In intervention villages, 509 (76.5%) caregivers at endline confirmed CFM-related activities had taken place in their village compared to only 39 (6.2%) caregivers in control villages. Almost half (306; 46.0%) of intervention caregivers reported their household attended all five activities while about one-fifth (126; 18.9%) did not attend any (Table S1). Most intervention caregivers reported receiving hardware with 378 (56.8%) having a latrine training mat, 62 (9.3%) a bucket with lid, and 56 (8.4%) a wash basin. About one-fifth (143; 21.5%) of intervention caregivers reported not receiving any hardware although their child was age eligible, but among these most did not attend any activities (109; 76.2%). Among caregivers who received the latrine training mat, 285 (75.4%) reported a desirable hardware practice: 98 (25.9%) were currently using it, 139 (36.8%) used it in the past but stopped because their child learned to use the latrine directly, and 48 (12.7%) never used it because their child already used the latrine (Table S2). Among those who received a bucket with lid or wash basin, 53 (85.5%) and 55 (98.2%), respectively, reported a desirable hardware practice: currently using it (37; 59.7% and 38; 67.9%), stopped using it because their child stopped defecating on cloths (5; 8.1% and 7; 12.5%), or were using another version of a similar hardware (11; 17.7% and 10; 17.9%). Overall, 447 (67.2%) intervention caregivers met the per-protocol criteria, which represented 576 children (Table S1).

### Primary analysis

In the fully adjusted ITT models, the prevalence of JMP-defined safe disposal was 1.16 times greater (95% CI 1.04–1.29) in the intervention arm compared to control at endline (Table 3). The prevalence of caregiver safe disposal was 1.46 times greater (95% CI 1.12–1.92), but there was no difference in prevalence for child latrine use at endline (PR 1.06; 95% CI 0.95–1.18).

**Table 3:** Effect of intervention on JMP-defined safe child feces disposal, caregiver safe disposal, and child latrine use

		Endline						Prevalence ratio†						
		*Intervention*			*Control*			*Unadjusted*				*Fully adjusted*§		
		N	n (%)		N	n (%)		PR	95% CI	*P* value		PR	95% CI	*P* value
JMP-defined safe child feces disposal*														
All children aged < 6 years^+^		840	653 (77.7%)		785	517 (65.9%)		1.21	1.05–1.39	**0.009**		1.16	1.04–1.29	**0.010**
Male children aged < 6 years		437	336 (76.9%)		405	271 (66.9%)		1.18	1.01–1.39	**0.042**		1.13	1.00–1.28	0.059
Female children aged < 6 years		403	317 (78.7%)		380	246 (64.7%)		1.22	1.05–1.41	**0.008**		1.17	1.04–1.32	**0.010**
Per-protocol		576	476 (82.6%)		785	517 (65.9%)		1.28	1.11–1.48	**0.001**		1.23	1.09–1.38	**0.001**
Children aged < 3 years		428	289 (67.5%)		405	189 (46.7%)		1.51	1.22–1.88	**< 0.001**		1.42	1.21–1.67	**< 0.001**
**Caregiver safe disposal**														
All children aged < 6 years		840	156 (18.6%)		785	107 (13.6%)		1.47	1.09–1.98	**0.012**		1.46	1.12–1.92	**0.006**
Male children aged < 6 years		437	80 (18.3%)		405	58 (14.3%)		1.37	0.93–2.03	0.109		1.40	0.96–2.03	0.079
Female children aged < 6 years		403	76 (18.9%)		380	49 (12.9%)		1.47	1.06–2.06	**0.023**		1.43	1.06–1.92	**0.018**
Per-protocol		576	107 (18.6%)		785	107 (13.6%)		1.45	1.05–2.00	**0.022**		1.44	1.07–1.93	**0.015**
Children aged < 3 years		428	148 (34.6%)		405	104 (25.7%)		1.45	1.09–1.95	**0.012**		1.44	1.11–1.86	**0.005**
**Child latrine use**														
All children aged < 6 years		842	497 (59.0%)		786	410 (52.2%)		1.12	0.96–1.30	0.158		1.06	0.95–1.18	0.332
Male children aged < 6 years		439	256 (58.3%)		406	213 (52.5%)		1.10	0.93–1.32	0.274		1.07	0.94–1.21	0.301
Female children aged < 6 years		403	241 (59.8%)		380	197 (51.8%)		1.13	0.96–1.33	0.144		1.08	0.94–1.24	0.284
Per-protocol		577	369 (64.0%)		786	410 (52.2%)		1.22	1.05–1.41	**0.009**		1.18	1.04–1.33	**0.010**
Children aged < 3 years		429	141 (32.9%)		406	85 (20.9%)		1.52	1.11–2.08	**0.009**		1.41	1.08–1.83	**0.012**

Bolded *P* values represent a significant result (significance *P* < 0.05)

^*^Safe child feces disposal, as defined by JMP, includes both latrine use by the child and safe disposal of child feces into a latrine by the caregiver. ^+^Children were < 5 years old at the start of intervention delivery (December 2021) but by the time of endline data collection, approximately four to six months later, some children reached 5 years old. †Models were log-binomial and used generalized estimating equations (GEE) with robust standard errors to account for clustering. However, all of the fully adjusted models for JMP-defined safe disposal and caregiver safe disposal, as well as the fully adjusted per-protocol model for child latrine use, did not converge. In this case, a Poisson model was used rather than log-binomial. §Fully adjusted models were adjusted for two village-level variables: geo-demo group (the variable used for stratified randomization) and baseline prevalence of the outcome

When restricted to the per-protocol sample, the prevalence of JMP-defined safe disposal slightly improved among the intervention arm compared to control (PR 1.23; 95% CI 1.09–1.38). The prevalence ratio for caregiver safe disposal remained relatively the same at 1.44 times greater (95% CI 1.07–1.93), while there was a significant difference in child latrine use with 1.18 times greater prevalence in the intervention arm compared to control (95% CI 1.04–1.33).

All unadjusted and partially adjusted (only geo-demo group) models gave similar results to the fully adjusted models (Table 3 and Table S3).

### Secondary analyses

No major differences in effects were found between girls and boys (Table 3), but differences were noted by child age. When the fully adjusted ITT models were restricted to children < 3 years old (Table 3), the prevalence ratio of JMP-defined safe disposal was much higher at 1.42 times greater prevalence in the intervention arm compared to control (95% CI 1.21–1.67). Similar improvements were found for both caregiver safe disposal (PR 1.44; 95% CI 1.11–1.86) and child latrine use (PR 1.41; 95% CI 1.08–1.83).

Child age was further examined by specific child age groups (Table S4). There was a significant difference in prevalence of JMP-defined safe disposal between intervention and control for three younger age groups, all of which align with motor development milestones: 8 to 11 months (master crawling), 12 to 17 months (master walking), and 24 to 35 months (master squatting). The significant difference for the younger two age groups mostly derived from higher prevalence of caregiver safe disposal while the older age group derived from higher prevalence of child latrine use. Caregiver safe disposal was 29.1 percentage points (95% CI 10.9–47.3) greater at endline in the intervention arm compared to control for children 8 to 11 months old and 15.2 percentage points greater (95% CI 0.2–30.6) for children 12 to 17 months old. Child latrine use was 20.7 percentage points greater (95% CI 6.7–34.7) for children 24 to 35 months old. Consequently, the mean age (in months) among children who used the latrine was significantly younger by about two to three months in the intervention arm compared to the control arm (mean difference: -2.37 months; 95% CI -4.42 – -0.31).

For secondary behavioral outcomes, we compared prevalence of safe practices along the CFM exposure pathway at endline (Table 4). There was a significant difference between intervention and control for the initial behavioral steps of the pathway but not the later steps. Child defecation on a safe material (i.e. non-porous and cleanable) was 9.6 percentage points (95% CI 1.4–17.9) higher in the intervention arm compared to the control arm. Congruently, child defecation directly on the ground in/near the household was 16.2 percentage points (95% CI -23.3 – -9.1) lower in the intervention arm compared to control. Caregiver use of a safe material to pick up the feces was 11.9 percentage points (95% CI 0.8–22.9) higher among intervention caregivers compared to control. For almost all remaining safe practices in the pathway — management of material used, child anal cleansing, and handwashing — the intervention arm had a higher prevalence but none were significantly different from the control arm. In addition, there was no difference between intervention and control for latrine use among children 6 to 10 years old, visibility of feces in the household compound, caregiver received social support, or caregiver perceived workload (Table S5).

**Table 4:** Comparison of prevalence for secondary CFM behaviors between intervention and control children at endline

	Intervention			Control			Difference
	*N*	*n* (%)		*N*	*n* (%)		Percentage points (95% CI)
Safe practices along CFM exposure pathway*							
**Step 1. Child defecates on safe material**							
Child defecated on non-porous material that can be cleaned	347	111 (32.0%)		376	84 (22.3%)		**9.6% (1.4 to 17.9)** ^**+**^
Child defecated directly on ground in/near household	347	121 (34.9%)		376	192 (51.1%)		**-16.2% (-23.3 to -9.1)**
**Step 2. Caregiver picks up feces with safe material**							
Caregiver used non-porous material to handle feces	342	181 (52.9%)		375	154 (41.1%)		**11.9% (0.8 to 22.9)** ^**+**^
***Step 3. Caregiver safely disposes of feces into latrine***	*See* Table 3						
**Step 4. Caregiver safely manages material used to pick up feces (washes with water and soap, disposes in latrine)**							
If used non-porous material, caregiver washed with water and soap/disinfectant	159	147 (92.5%)		132	117 (88.6%)		3.8% (-13.7 to 21.4)
Caregiver then disposed of wash water into latrine	157	89 (56.7%)		129	59 (45.7%)		11.0% (-16.5 to 38.4)
If used cloth specifically, caregiver washed soiled cloth in a dedicated wash basin/container	125	82 (65.6%)		120	79 (65.8%)		-0.2% (-28.6 to 28.1)
If not immediately cleaned, caregiver stored soiled cloths in bucket/container with lid	48	17 (35.4%)		46	9 (19.6%)		15.9% (-38.7 to 70.4)
If used porous, biodegradable material (leaves, straw, or paper), caregiver disposed of material into latrine	106	30 (28.3%)		164	19 (11.6%)		16.7% (-18.0 to 51.5)
**Step 5. Child anal cleansing is done in safe location**†							
Child bottom cleaned in latrine over pan or over latrine mat with tray or in bucket	841	439 (52.2%)		785	375 (47.8%)		4.4% (-4.5 to 13.3)
**Step 6. Caregiver and child wash hands with water and soap**							
Caregiver washed hands with water and soap	647	588 (90.9%)		605	529 (87.4%)		3.4% (-1.9 to 8.8)
Child’s hands were washed with water and soap	345	207 (60.0%)		376	200 (53.2%)		6.8% (-3.6 to 17.2)
Child washed hands after defecating in latrine	497	452 (91.0%)		409	369 (90.2%)		0.7% (-5.9 to 7.3)
**Consistency of safe practice in last week** §							
Child’s feces always ended up in latrine (caregiver safely disposed and/or child used latrine)	828	604 (73.0%)		778	487 (62.6%)		10.4% (-0.5 to 21.2)
Safe material always used to handle child’s feces	342	165 (48.3%)		375	136 (36.3%)		**12.0% (1.2 to 22.8)**

*Practices are self-reported by caregiver and capture how the child’s feces were managed the *last time* the child defecated. Steps 1 to 3 are CFM practices reported on for children who did not use the latrine; steps 4 and 5 are hygiene practices reported on for all children. ^+^Difference here can be attributed to intervention caregivers using the latrine mat with tray (*n* = 26) because the same proportion of caregivers in both study arms used each of the other safe (i.e. non-porous) materials. Safe materials included latrine mat with tray, only tray, cloth, child’s clothing, diapers, potty, shovel, hoe, and dustpan. In both study arms, cloth was the most common safe material used for what the child defecated on and to handle the feces. †Only 2 children did not have anal cleansing done after defecation. § After being asked about the last time the child defecated, caregivers were asked where else the child defecated and what other materials were used to handle the child’s feces in the past week in order to assess consistency of a safe practice

### Mediation analysis

**Fig. 2:**
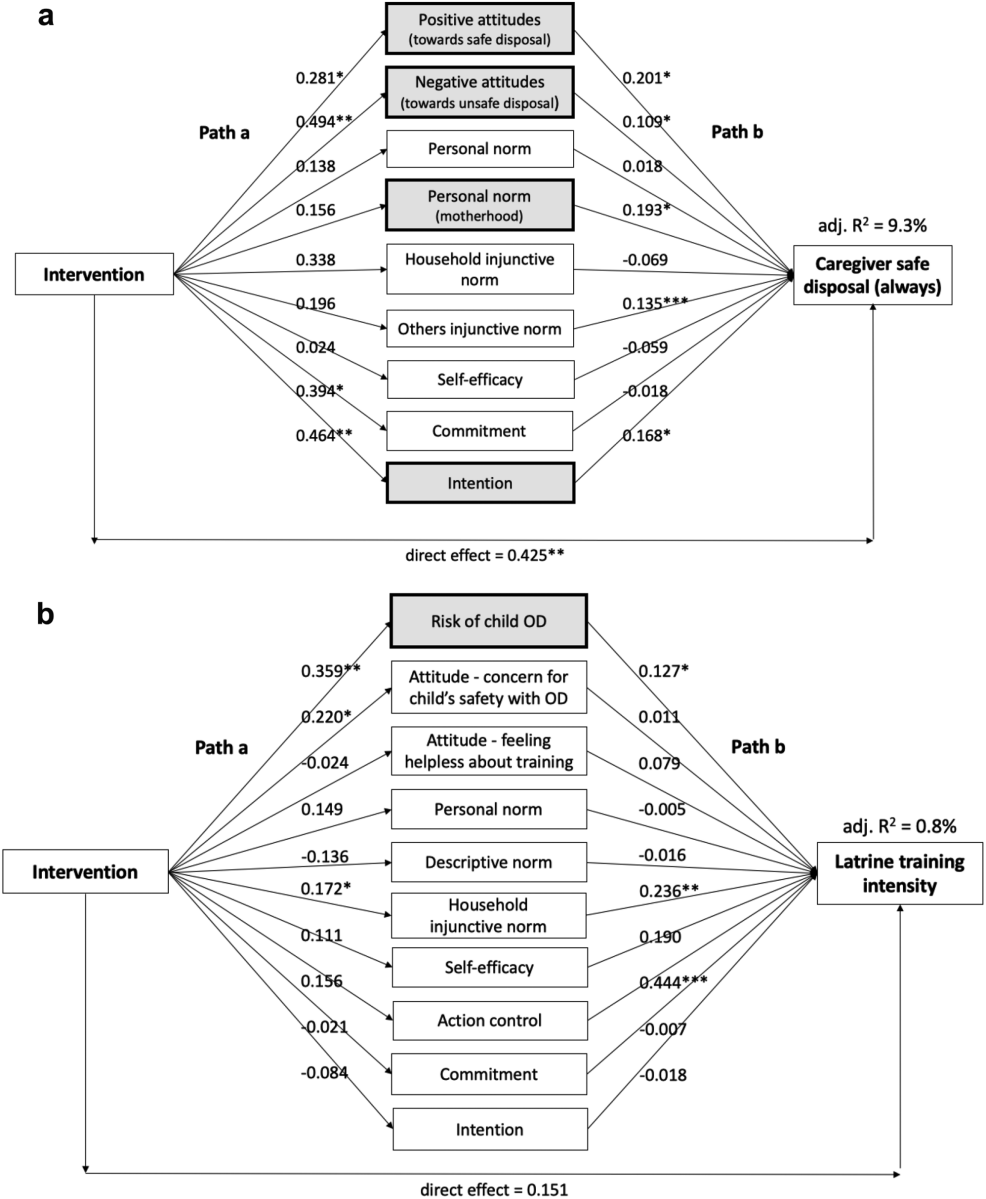
**(a)** Mediation analysis of psychosocial factors associated with intervention and caregiver safe disposal consistency. **(b)** Mediation analysis of psychosocial factors associated with intervention and child latrine training intensity^+^. Path a numbers represent unstandardized coefficients of single regression models measuring the association between the intervention and each psychosocial factor. Path b numbers represent unstandardized coefficients of multiple regression models measuring the association between the psychosocial factors and the behavioral outcome. Shaded boxes represent psychosocial factors that had significant indirect effects (ab), indicating these factors mediated the effect of the intervention on the behavioral outcome; *p < .05; **p < .01; ***p < .001. ^+^Latrine training intensity was measured by six-point Likert. Caregivers were asked, “During the last week, when your child needed to defecate, how often did you take your child to the latrine and teach them how to use it?” with responses from 0 (never) to 5 ((almost) always (100%))

The mediation results for caregiver safe disposal consistency are presented in Fig. 2a. The intervention’s effect on caregiver safe disposal was mediated by four psychosocial factors: positive attitudes towards safe disposal, negative attitudes towards unsafe disposal, personal norm that safe disposal is part of being a good mother, and intention to safely dispose. Along with these four factors, caregiver safe disposal was also associated with an injunctive norm that others approve of safe disposal. However, the intervention was not significantly associated with this factor, but was associated with stronger commitment towards safe disposal.

The mediation results for child latrine training intensity are presented in Fig. 2b. The intervention’s effect on latrine training intensity was mediated only by perceived risk of child open defecation (OD). Latrine training intensity was associated with this risk factor as well as action control around latrine training and a household injunctive norm that one’s child is expected to be taught how to use the latrine. The intervention was not associated with greater action control but was associated with a stronger household injunctive norm, although this did not mediate the effect on latrine training intensity, as well as greater concern for child’s safety when open defecating.

### Sensitivity analysis

We compared models stratified by caregivers who were surveyed twice (baseline and endline) versus once (endline) to determine if the intervention had differential effects on these two populations (Table S6). Out of the caregivers surveyed at endline, 314 caregivers (46.9%) in the intervention arm and 291 caregivers (45.6%) in the control arm had also participated in the baseline survey. For JMP-defined safe disposal and child latrine use, model results were similar for both groups. For caregiver safe disposal, prevalence was much higher in the intervention arm compared to control for those surveyed twice (surveyed twice PR 1.98, 95% CI 1.14–3.45; surveyed once PR 1.27, 95% CI 0.94–1.71).

## Discussion

We assessed the effect of a combined behavior change and hardware intervention on safe child feces disposal among children < 5 years old at the time of the intervention. In our primary analysis, intention-to-treat results showed a significant effect on JMP-defined safe disposal, with improvements in caregiver safe disposal but no effect on child latrine use. When the analysis was restricted to children < 3 years old, we found the intervention had a larger effect on both caregiver safe disposal and child latrine use, with a two-fifths greater prevalence of each behavior in the intervention arm compared to control. Comparisons by child age group further illustrated the intervention significantly improved caregiver safe disposal among babies and younger toddlers aged 8 to 17 months, while child latrine use significantly improved among toddlers aged 24 to 35 months. Overall, the intervention increased caregiver safe disposal among those age groups with some of the lowest safe disposal rates at baseline and also increased child latrine use among the exact age group that is developmentally ready to toilet train [9–11].

While increasing safe CFM practices to a level that may yield health benefits has been challenging, this intervention may have achieved the target. Research suggests community coverage of improved child feces disposal must reach 75% or more to effect health, such as reduce child stunting [6]. This may partly explain why other trials of sanitation interventions reported mixed effects on diarrhea and no effect on child stunting [26, 27]. The WASH-Benefits trial in Kenya only reached 33–37% safe disposal in the water, sanitation and hygiene (WASH) study arms while the Sanitation Hygiene Infant Nutrition Efficacy (SHINE) trial in Zimbabwe did reach 77% prevalence in their WASH study arm, but it was specifically for safe disposal of wash water from cleaning soiled nappies among *infants* [26, 27]. Similar to our intervention, both the WASH-Benefits and SHINE intervention activities were developed based on rigorous formative research, behavioral theory, and piloting. However, the WASH-Benefits intervention attempted to address a variety of WASH behaviors rather than focus activities solely on CFM practices, which may have led to the lower behavioral adoption of safe disposal. The SHINE intervention achieved high adoption of safe disposal, but it was specifically for infant feces and did not address safe CFM practices for toddlers and young children. Caruso et al. (2022) evaluated the effect of a sanitation behavior change intervention in Odisha ─ named *Sundara Grama* — that promoted safe CFM through mothers groups and provision of plastic potties and scoops [22]. The intervention had greater impact on caregiver safe disposal (20.4 percentage point increase) than child latrine use (7.1 percentage point increase) when examining all children < 5 years old, but also showed particular improvements in caregiver safe disposal among younger toddlers and improvements in child latrine use among older toddlers. The *Sundara Grama* intervention was very similar to our intervention: rigorously developed, focused on latrine use behaviors, and addressed CFM practices across child age groups. However, the intervention included only one activity dedicated to CFM behavior change — the mothers group meeting. The intervention achieved 60.0% prevalence of JMP-defined safe disposal [22]. Our intervention achieved 77.7% prevalence at endline, suggesting the intervention could positively impact child health.

The differential effects of the intervention on caregiver safe disposal compared to child latrine use among all children may be a result of underlying differences between these two distinct behaviors. First, caregiver safe disposal and child latrine use are relevant to different child age groups and greatly differed in their baseline prevalence; as such, this creates disproportionate opportunities for behavioral improvement. Caregiver safe disposal was rare across all age groups (9.6% baseline prevalence) while child latrine use was already common among older children ages 3 to 5 years old (69.6% baseline prevalence), leaving mostly the 2 to 3-year-old group to improve. The diffusion of innovation theory explains it is harder to spark behavioral change among “late adopters,” who are more skeptical of change, than “early adopters” [28]. When examining the full sample of children, the intervention may have had no effect on child latrine use because most older children already used the latrine and those who did not could be viewed as “late adopters.” In contrast, when the sample was restricted to children < 3 years old, there was a greater opportunity to improve latrine use, especially among 2 to 3-year-olds, and with more “early adopters.”

Second, the mediation results suggest the intervention was more successful at targeting psychosocial factors steering caregiver safe disposal than factors steering child latrine training. The intervention had an indirect effect on caregiver safe disposal through influencing attitudes, norms, and behavioral intention but only had an indirect effect on child latrine training through influencing risk perceptions of child open defecation. The intervention activities were designed to target a variety of psychosocial factors for larine training, but this was not achieved. It could be that influencing caregivers’ risk perceptions of child open defection was compelling enough to motivate latrine training for caregivers of younger toddlers under 3 years old, but other psychosocial factors needed to be more effectively targeted for improving latrine use among households with “late adopter” 3 to 5-year-olds.

The successful impact of the intervention on *both* caregiver safe disposal and child latrine use among children < 3 years old may stem in part from the novel latrine training mat and “transitions” messaging. The latrine training mat was specifically designed to “grow” with the child and in this way acted as a single hardware that aided *both* behaviors. Importantly, this novel hardware was embedded into behavior change activities to ensure appropriate adoption, and activities emphasized behavioral messaging on when and how caregivers should navigate the transition to latrine training with their child and strategies for overcoming challenges. Other interventions have encouraged the use of plastic potties but report limited uptake and mixed reviews [14, 29, 30]. Depending on the sanitation context, potties may only serve as a caregiver safe disposal hardware and actually hinder progress towards latrine use as potties teach children to sit rather than squat to defecate. Child toilet training is a particularly “missed opportunity” in WASH interventions to eliminate fecal exposure. While the Indian Academy of Pediatrics advices children are developmentally ready to toilet train around 2 years old [9], nationally only 25.3% of Indian children aged 24 to 35 months used a latrine at last defecation [31]. And as children get older, their feces are more likely to be left in the open if they defecate outside the latrine [8].

The intervention led to improvements beyond safe disposal and child latrine use alone, but in other safe CFM behaviors as well since messaging addressed the full “CFM exposure pathway.” Significantly more children in the intervention arm defecated on a safe material, rather than on the ground, and had their feces picked up/handled with a safe material; much of which can be attributed to caregivers using the latrine training mat with tray. Interventions to date, such as the SHINE and WASH-Benefits interventions, often target a variety of WASH behaviors at once but only address a particular CFM practice for a specific age group, which may be another reason for their limited health impacts [26, 27, 32].

The COVID-19 pandemic introduced a number of study limitations. During the intervention design stage with Gram Vikas, it was decided to not include community-wide activities in order to minimize large gatherings. The intervention was also implemented in a shorter timeframe to ensure delivery took place during a window of low COVID case counts. Study activities were paused twice for several months due to the pandemic, and resulted in endline data collection taking place approximately 2.5 years after baseline. Because of this, many children at baseline “aged out” of the study by intervention delivery so it was not possible to measure true change in behavior among a single caregiver sample. We planned to measure fecal contamination of household drinking water and childs’ hands at endline [15] but did not pursue this sampling collection because it would require close contact with participants. In addition to these COVID-related limitations, the target behaviors were measured by caregiver self-report, a possible source of bias that we aimed to mitigate by following recommended practices [33]. COVID and other constraints also limited our ability to assess the longer-term adoption of the CFM practices promoted by the intervention. Lastly, the findings from this trial are likely not generalizable to contexts with poor WASH access as we specifically engaged caregivers who had a household latrine and many also had piped water supply. However, this may in fact be the more generalizable population to engage in CFM studies as global gains to sanitation access continue.

## Conclusions

Calls have been made for transformative WASH approaches that drastically reduce fecal contamination in the household environment in order to realize health impacts: safe CFM practices are fundamental to such an approach [34]. The Government of India proclaimed the country open defecation free (ODF), but child feces remain poorly managed with almost no mention of CFM in the current national sanitation scheme (Swachh Bharat Mission phase II) [35]. Our behavior change and hardware intervention led to significant increases in caregiver safe disposal and child latrine use among children < 3 years old, and improved other safe CFM behaviors. While future research is needed to demonstrate sustainability of these effects, our results suggest a potentially scalable intervention for improving child feces disposal and reducing disease. The intervention’s success at improving safe CFM behaviors may be attributed to several intervention elements: effective behavior change techniques for caregiver safe disposal, provision of the novel latrine training mat hardware, promotion of fully safe CFM practices, and a child development lens with messaging on transitions. We recommend the Government of India, as well as future ‘transformative WASH’ investments, incorporate and build upon these critical intervention elements to foster healthier households.

### Electronic supplementary material

Below is the link to the electronic supplementary material.

Supplementary Material 1

Supplementary Material 2

## Data Availability

The raw data and corresponding codebook used in the analysis are available upon reasonable request from the corresponding author GDS at gloria.sclar@emory.edu.

## Abbreviations

CFM: Child feces management
MANTRA: Movement and Action Network for Transformation of Rural Areas
RANAS: Risks, Attitudes, Norms, Ability, Self-regulation
BCT: Behavior change technique
UCD: User-centered design
ODK: Open Data Kit
JMP: WHO-UNICEF Joint Monitoring Program on Water, Sanitation and Hygiene
ICC: Intra-class correlation
PR: Prevalence ratio
ITT: Intention-to-treat
GEE: Generalized estimating equations
WASH: Water, sanitation, and hygiene
SHINE: Sanitation Hygiene Infant Nutrition Efficacy (trial)
