# Supplementary material for: Effect of a behavior change and hardware intervention on safe child feces management practices in rural Odisha, India: a cluster-randomized controlled trial
Source: BMC Public Health. 2024 Aug 27;24:2327. doi: 10.1186/s12889-024-19272-5 (PMC11351010; doi:10.1186/s12889-024-19272-5)

**SUPPLEMENTAL File 1**

**Title:** Effect of a behavior change and hardware intervention on safe child feces management practices in rural Odisha, India: a cluster-randomized controlled trial

**Table S1.** Self-reported attendance at intervention activities and hardware received

|  | **Intervention Caregivers**  **(N = 665)^*^** | |
| --- | --- | --- |
| **Attendance by activity** | **n** | **%** |
| Opening hardware meeting | 502 | 75.5% |
| Household visits^+^ | 455 | 68.4% |
| Caregiver meeting | 451 | 67.8% |
| Closing celebratory meeting | 417 | 62.7% |
| **Number of total activities attended** |  |  |
| 0 | 126 | 18.9% |
| 1 | 29 | 4.4% |
| 2 | 30 | 4.5% |
| 3 | 72 | 10.8% |
| 4 | 95 | 14.3% |
| 5 | 306 | 46.0% |
| **Hardware received** |  |  |
| Latrine training mat | 356 | 53.5% |
| Wash basin and bucket w/ lid | 35 | 5.3% |
| All 3 | 21 | 3.2% |
| Only bucket w/ lid | 5 | 0.8% |
| Latrine training mat and bucket w/ lid | 1 | 0.2% |
| None - child was >= 4 years old | 96 | 14.4% |
| None - child was <4 years old | 143 | 21.5% |
| **Per-protocol sample** |  |  |
| Attended at least 3 activities and received hardware (if child <4 years old) | 447 | 67.2% |

^*^7 caregivers ended the survey early before answering the intervention activity questions and 5 caregivers ended before the hardware received question. ^+^390 caregivers (58.6%) reported attending both household visits and 65 (9.7%) reported only attending one household visit

**Table S2.** Caregiver reported hardware use

| **Hardware use** | **Bucket with lid**  **(to store soiled cloths)** | | **Wash basin**  **(to wash soiled cloths)** | | **Latrine training mat** | |
| --- | --- | --- | --- | --- | --- | --- |
|  | N = 62 | | N = 56 | | N = 378 | |
|  | n | % | n | % | n | % |
| ***Desirable hardware use or acceptable non-use**** | **53** | **85.5%** | **55** | **98.2%** | **285** | **75.4%** |
| Currently used in past week | 37 | 59.7% | 38 | 67.9% | 98 | 25.9% |
| Used in the past but transitioned behavior^+^ | 5 | 8.1% | 7 | 12.5% | 139 | 36.8% |
| Never used because child already used latrine | 0 | 0.0% | 0 | 0.0% | 48 | 12.7% |
| Used another version of similar hardware† | 11 | 17.7% | 10 | 17.9% | 0 | 0.0% |
| ***Did not use hardware or stopped using for undesirable reason*** | **9** | **14.5%** | **1** | **1.8%** | **93** | **24.6%** |
| Used in the past but hardware broke | 4 | 6.5% | 0 | 0.0% | 0 | 0% |
| Used in the past but stopped for other reason | 3 | 4.8% | 1 | 1.8% | 52 | 13.8%^§^ |
| Never used for other reason | 2 | 3.2% | 0 | 0% | 41 | 10.8%^§^ |

*Hardware was intended to be a short-term tool to facilitate either safe storage and washing of soiled cloths, or safe latrine training, so it is considered desirable for a child to transition to the next child development stage related-CFM behavior or to not need to use a hardware because they are already using the latrine directly.

^+^For bucket and basin, this means child no longer defecates on cloth; for latrine mat this means child started using the latrine directly without needing the latrine mat

†This refers to a different bucket or wash basin than the one provided by the intervention being used for storing or cleaning soiled cloths. For example, caregivers may have washed soiled cloths in the provided bucket directly instead of washing in a wash basin, or caregivers may have stored soiled cloths in another bucket with lid that they already owned, so the specific provided hardware was not needed.

^§^The two most common other reasons given were that the child refused to use the latrine mat, or that the household has a lack of water which prevented them from using the latrine mat.

**Table S3.** Partially adjusted models for ITT, sex-stratified, and PP analyses for each behavior

|  |  | **Prevalence ratio**† | | | | |
| --- | --- | --- | --- | --- | --- | --- |
|  |  |  | **Adjusted for geo-demo**§ | | | |
|  |  |  | N | PR | 95% CI | p value |
| **JMP-defined safe child feces disposal^*^** |  |  |  |  |  |  |
| All children aged <6 years^+^ |  |  | 1625 | 1.20 | 1.06 – 1.36 | **0.004** |
| Male children aged <6 years |  |  | 842 | 1.17 | 1.03 – 1.34 | **0.017** |
| Female children aged <6 years |  |  | 783 | 1.22 | 1.06 – 1.39 | **0.004** |
| Per-protocol |  |  | 1361 | 1.29 | 1.13 – 1.48 | **<0.001** |
| Children aged <3 years |  |  | 833 | 1.48 | 1.22 – 1.80 | **<0.001** |
| **Caregiver safe disposal** |  |  |  |  |  |  |
| All children aged <6 years |  |  | 1625 | 1.46 | 1.12 – 1.92 | **0.006** |
| Male children aged <6 years |  |  | 842 | 1.39 | 0.96 – 2.03 | 0.081 |
| Female children aged <6 years |  |  | 783 | 1.43 | 1.07 – 1.93 | **0.016** |
| Per-protocol |  |  | 1361 | 1.44 | 1.08 – 1.93 | **0.013** |
| Children aged <3 years |  |  | 833 | 1.42 | 1.10 – 1.84 | **0.007** |
| **Child latrine use** |  |  |  |  |  |  |
| All children aged <6 years |  |  | 1628 | 1.11 | 0.96 – 1.29 | 0.164 |
| Male children aged <6 years |  |  | 845 | 1.11 | 0.94 – 1.32 | 0.215 |
| Female children aged <6 years |  |  | 783 | 1.12 | 0.95 – 1.32 | 0.163 |
| Per-protocol |  |  | 1363 | 1.21 | 1.05 – 1.40 | **0.007** |
| Children aged <3 years |  |  | 835 | 1.50 | 1.12 – 2.00 | **0.006** |

JMP=WHO/UNICEF Joint Monitoring Programme for Water Supply, Sanitation, and Hygiene. ^*^Safe child feces disposal, as defined by JMP, includes both latrine use by the child and safe disposal of child feces into a latrine by the caregiver. ^+^Children were <5 years old at the start of intervention delivery (December 2021) but by the time of endline data collection, approximately four to six months later, some children reached 5 years old. †Models were log-binomial and used generalized estimating equations (GEE) with robust standard errors to account for clustering. However, the per-protocol models for JMP-defined safe disposal and caregiver safe disposal did not converge and Poisson was used instead of log-binomial. §Models were adjusted only for geo-demo group (village-level variable used for stratified randomization).

**Table S4.** Comparisons of prevalence for each behavior by child-age group between intervention and control at endline

|  | **Baseline Prevalence** | | | | |  | **Endline Prevalence** | | | | |  | **Endline difference** | **95% CI** |
| --- | --- | --- | --- | --- | --- | --- | --- | --- | --- | --- | --- | --- | --- | --- |
|  | Intervention  (n = 562) | |  | Control  (n = 542) | |  | Intervention  (n = 840) | |  | Control  (n = 785) | |  | (percentage points) |  |
|  | N | n (%) |  | N | n (%) |  | N | n (%) |  | N | n (%) |  |  |  |
| **JMP-defined safe child feces disposal** |  |  |  |  |  |  |  |  |  |  |  |  |  |  |
| 0 to 7 months | 57 | 23 (40.4%) |  | 59 | 17 (28.8%) |  | 90 | 50 (55.6%) |  | 92 | 42 (45.7%) |  | 9.9% | -8.3 – 28.1 |
| 8 to 11 months | 30 | 11 (36.7%) |  | 38 | 11 (29.0%) |  | 55 | 36 (65.5%) |  | 55 | 19 (34.6%) |  | **30.9%** | **10.5 – 51.3** |
| 12 to 17 months | 54 | 11 (20.4%) |  | 47 | 7 (14.9%) |  | 71 | 42 (59.2%) |  | 60 | 19 (31.7%) |  | **27.5%** | **8.3 – 46.6** |
| 18 to 23 months | 66 | 20 (30.3%) |  | 56 | 14 (25.0%) |  | 67 | 47 (70.2%) |  | 76 | 40 (52.6%) |  | 17.5% | -1.6 – 36.6 |
| 24 to 35 months | 108 | 56 (51.9%) |  | 120 | 59 (49.2%) |  | 145 | 114 (78.6%) |  | 122 | 69 (56.6%) |  | **22.1%** | **6.9 – 37.2** |
| 36 to 47 months | 135 | 87 (64.4%) |  | 116 | 79 (68.1%) |  | 160 | 134 (83.8%) |  | 145 | 118 (81.4%) |  | 2.4% | -9.4 – 14.1 |
| 48 to 59 months | 112 | 86 (76.8%) |  | 106 | 81 (76.4%) |  | 147 | 131 (89.1%) |  | 126 | 109 (86.5%) |  | 2.6% | -7.7 – 12.9 |
| 60 to 69 months | - | - |  | - | - |  | 105 | 99 (94.3%) |  | 109 | 101 (92.7%) |  | 1.6% | -7.0 – 10.3 |
| **Caregiver safe disposal** |  |  |  |  |  |  |  |  |  |  |  |  |  |  |
| 0 to 7 months | 57 | 23 (40.4%) |  | 59 | 17 (28.8%) |  | 90 | 49 (54.4%) |  | 92 | 41 (44.6%) |  | 9.9% | -5.1 – 24.9 |
| 8 to 11 months | 30 | 10 (33.3%) |  | 38 | 10 (26.3%) |  | 55 | 34 (61.8%) |  | 55 | 18 (32.7%) |  | **29.1%** | **10.9 – 47.3** |
| 12 to 17 months | 54 | 8 (14.8%) |  | 47 | 4 (8.5%) |  | 71 | 25 (35.2%) |  | 60 | 12 (20.0%) |  | **15.2%** | **0.2 – 30.6** |
| 18 to 23 months | 66 | 10 (15.2%) |  | 56 | 5 (8.9%) |  | 67 | 19 (28.4%) |  | 76 | 17 (22.4%) |  | 6.0% | -8.8 – 20.7 |
| 24 to 35 months | 108 | 4 (3.7%) |  | 120 | 9 (7.5%) |  | 145 | 21 (14.5%) |  | 122 | 16 (13.1%) |  | 1.4% | -7.4– 10.2 |
| 36 to 47 months | 135 | 0 (0%) |  | 116 | 3 (2.6%) |  | 160 | 6 (3.8%) |  | 145 | 2 (1.4%) |  | 2.4% | -1.4 – 6.1 |
| 48 to 59 months | 112 | 2 (1.8%) |  | 106 | 1 (0.9%) |  | 147 | 2 (1.4%) |  | 126 | 1 (0.8%) |  | 0.6% | -2.0 – 3.1 |
| 60 to 69 months | - | - |  | - | - |  | 105 | 0 (0%) |  | 109 | 0 (0%) |  | 0% | - |
| **Child latrine use** |  |  |  |  |  |  |  |  |  |  |  |  |  |  |
| 0 to 7 months | 57 | 0 (0%) |  | 59 | 0 (0%) |  | 91 | 1 (1.1%) |  | 92 | 1 (1.1%) |  | 0% | -3.4 – 3.4 |
| 8 to 11 months | 30 | 1 (3.3%) |  | 38 | 1 (2.6%) |  | 55 | 2 (3.6%) |  | 55 | 1 (1.8%) |  | 1.8% | -4.7 – 8.3 |
| 12 to 17 months | 54 | 3 (5.6%) |  | 48 | 3 (6.3%) |  | 71 | 17 (23.9%) |  | 60 | 7 (11.7%) |  | 12.3% | -1.6 – 26.2 |
| 18 to 23 months | 66 | 10 (15.2%) |  | 56 | 9 (16.1%) |  | 67 | 28 (41.8%) |  | 77 | 23 (29.9%) |  | 11.9% | -5.4 – 29.2 |
| 24 to 35 months | 110 | 52 (47.3%) |  | 121 | 50 (41.3%) |  | 145 | 93 (64.1%) |  | 122 | 53 (43.4%) |  | **20.7%** | **6.7 – 34.7** |
| 36 to 47 months | 136 | 87 (64.0%) |  | 116 | 76 (65.5%) |  | 160 | 128 (80.0%) |  | 145 | 116 (80.0%) |  | 0% | -10.7 – 10.7 |
| 48 to 59 months | 112 | 84 (75.0%) |  | 106 | 80 (75.5%) |  | 148 | 129 (87.2%) |  | 126 | 108 (85.7%) |  | 1.4% | -8.0 – 10.9 |
| 60 to 69 months | - | - |  | - | - |  | 105 | 99 (94.3%) |  | 109 | 101 (92.7%) |  | 1.6% | -6.0 – 9.2 |
| Mean age in months among children who used latrine (SD) |  |  |  |  |  |  | 497 | 44.90 (14.89) |  | 410 | 47.27 (14.17) |  | **-2.37 months** | **-4.42 – -0.31** |

**Table S5.** Comparison of additional secondary outcomes between intervention and control caregivers at endline

|  | **Intervention** | |  | **Control** | |  | **Difference^**^**  (95% CI) |
| --- | --- | --- | --- | --- | --- | --- | --- |
|  | N | n (%) |  | N | n (%) |  |  |
| **Received social support for child feces management: mean (SD)^+^** |  |  |  |  |  |  |  |
| Emotional support score | 146 | 3.54 (1.45) |  | 175 | 3.28 (1.36) |  | 0.26 (-0.06 to 0.58) |
| Informational support score | 147 | 3.54(1.74) |  | 176 | 3.56 (1.83) |  | -0.02 (-0.42 to 0.38) |
| Instrumental support score | 149 | 4.72 (1.25) |  | 178 | 4.57 (1.32) |  | 0.15 (-0.13 to 0.44) |
|  |  |  |  |  |  |  |  |
| **Received social support for child latrine training: mean (SD)^+^** |  |  |  |  |  |  |  |
| Emotional support score | 333 | 3.62 (1.46) |  | 294 | 3.49 (1.44) |  | 0.14 (-0.11 to 0.38) |
| Informational support score | 332 | 3.64 (1.86) |  | 290 | 3.49 (1.83) |  | 0.15 (-0.15 to 0.44) |
| Instrumental support score | 347 | 4.54 (1.37) |  | 297 | 4.58 (1.38) |  | -0.04 (-0.29 to 0.21) |
|  |  |  |  |  |  |  |  |
| **Latrine use among children 6 to 10 years old** | 378 | 327 (86.5%) |  | 341 | 304 (89.2%) |  | -2.6% (-15.5 to 10.2) |
|  |  |  |  |  |  |  |  |
| **Caregiver perceives her CFM workload as some to a great deal of work (vs. no to a little work)^±^** | 345 | 85 (24.6%) |  | 377 | 88 (23.3%) |  | -1.3% (-6.2 to 8.9) |
|  |  |  |  |  |  |  |  |
| **Caregiver perceives her child latrine training workload as some to a great deal of work (vs. no to little work) ^±^** | 282 | 56 (19.9%) |  | 212 | 34 (16.1%) |  | 3.8% (-11.3 to 18.9) |
|  |  |  |  |  |  |  |  |
| **Household contamination metrics** |  |  |  |  |  |  |  |
| Any feces visible in household compound^*^ | 594 | 114 (19.2%) |  | 562 | 120 (21.4%) |  | -2.2% (-9.5 to 5.1) |
| Human feces visible in household compound | 594 | 14 (2.4%) |  | 562 | 8 (1.4%) |  | 0.9% (-0.6 to 2.5) |
|  |  |  |  |  |  |  |  |

^+^Caregivers answered several questions that asked if they had experienced a specific supportive act with safe disposal or child latrine training in the last week with a Likert scale response from 1 (completely disagree) to 6 (completely agree). Questions were adapted from validated metrics and examined three types of support: emotional, informational, and instrumental support (16). Responses to questions related to each type of support were summed and then averaged to generate a score.

^±^Caregivers were asked “The last time your child defecated, how big or small of a task was it for you [to manage their feces OR to help them use the latrine]?” with a Likert scale response from 1 (no work) to 5 (a great deal of work).

^*^Enumerators noted what type of feces they observed in the household compound, if any, using the following four categories: oxen/cattle; goats/sheep; birds; human/pig/dog/monkey.

**The difference is reported as the difference in mean score for the social support outcomes and difference in percentage points for all other outcomes.

**Table S6.** Effect of intervention on safe disposal behaviors for caregivers surveyed twice and caregivers surveyed only at endline

|  | **Endline Prevalence** | | | | | | | | |  | **Prevalence Ratio**^+^ | | | | | | | | | | | | | | | | | | | |  |
| --- | --- | --- | --- | --- | --- | --- | --- | --- | --- | --- | --- | --- | --- | --- | --- | --- | --- | --- | --- | --- | --- | --- | --- | --- | --- | --- | --- | --- | --- | --- | --- |
|  | Intervention | | | |  | Control | | | |  | **Unadjusted** | | | | | |  | **Adjusted for geo-demo** | | | | | |  | **Fully adjusted**† | | | | | |  |
|  | | N | | n (%) | |  | N | | n (%) | |  | PR | | 95% CI | | p value | |  | PR | | 95% CI | | p value | |  | PR | | 95% CI | | p value | |
| **JMP-defined safe child feces disposal^*^** |  | |  | |  |  | |  | |  |  | |  | |  | |  |  | |  | |  | |  |  | |  | |  | |  |
| Caregiver surveyed only endline | 441 | | 320 (72.6%) | |  | 415 | | 248 (59.8%) | |  | 1.23 | | 1.05-1.44 | | **0.009** | |  | 1.23 | | 1.07 – 1.42 | | **0.005** | |  | 1.18 | | 1.03 – 1.34 | | **0.014** | |  |
| Caregiver surveyed twice | 399 | | 333 (83.5%) | |  | 370 | | 269 (72.7%) | |  | 1.15 | | 1.00-1.32 | | **0.047** | |  | 1.15 | | 1.02 – 1.30 | | **0.027** | |  | 1.12 | | 1.00 – 1.26 | | 0.060 | |  |
| **Caregiver safe disposal** |  | |  | |  |  | |  | |  |  | |  | |  | |  |  | |  | |  | |  |  | |  | |  | |  |
| Caregiver surveyed only endline | 441 | | 97 (22.0%) | |  | 415 | | 78 (18.8%) | |  | 1.25 | | 0.90 – 1.74 | | 0.190 | |  | 1.26 | | 0.94 – 1.69 | | 0.127 | |  | 1.27 | | 0.94 – 1.71 | | 0.119 | |  |
| Caregiver surveyed twice | 399 | | 59 (14.8%) | |  | 370 | | 29 (7.8%) | |  | 1.99 | | 1.13 – 3.50 | | **0.016** | |  | 2.03 | | 1.18 – 3.51 | | **0.011** | |  | 1.98 | | 1.14 – 3.45 | | **0.015** | |  |
| **Child latrine use** |  | |  | |  |  | |  | |  |  | |  | |  | |  |  | |  | |  | |  |  | |  | |  | |  |
| Caregiver surveyed only endline | 442 | | 223 (50.5%) | |  | 416 | | 170 (40.9%) | |  | 1.20 | | 0.98 – 1.47 | | 0.073 | |  | 1.20 | | 1.00 – 1.45 | | 0.058 | |  | 1.12 | | 0.97 – 1.28 | | 0.120 | |  |
| Caregiver surveyed twice | 400 | | 274 (68.5%) | |  | 370 | | 240 (64.9%) | |  | 1.05 | | 0.90 – 1.23 | | 0.505 | |  | 1.05 | | 0.91 – 1.22 | | 0.513 | |  | 1.03 | | 0.92 – 1.16 | | 0.591 | |  |

JMP=WHO/UNICEF Joint Monitoring Programme for Water Supply, Sanitation, and Hygiene. ^*^Safe child feces disposal, as defined by JMP, includes both latrine use by the child and safe disposal of child feces into a latrine by the caregiver. ^+^Models were log-binomial and used generalized estimating equations (GEE) with robust standard errors to account for clustering. However, some models did not converge and Poisson was used instead of log-binomial (for the fully adjusted models for JMP-defined safe disposal). †Fully adjusted models were adjusted for two village-level variables: geo-demo group (the variable used for stratified randomization) and baseline prevalence of the outcome.

**Figure S1.** Description, photos, and instructions for use for each intervention hardware


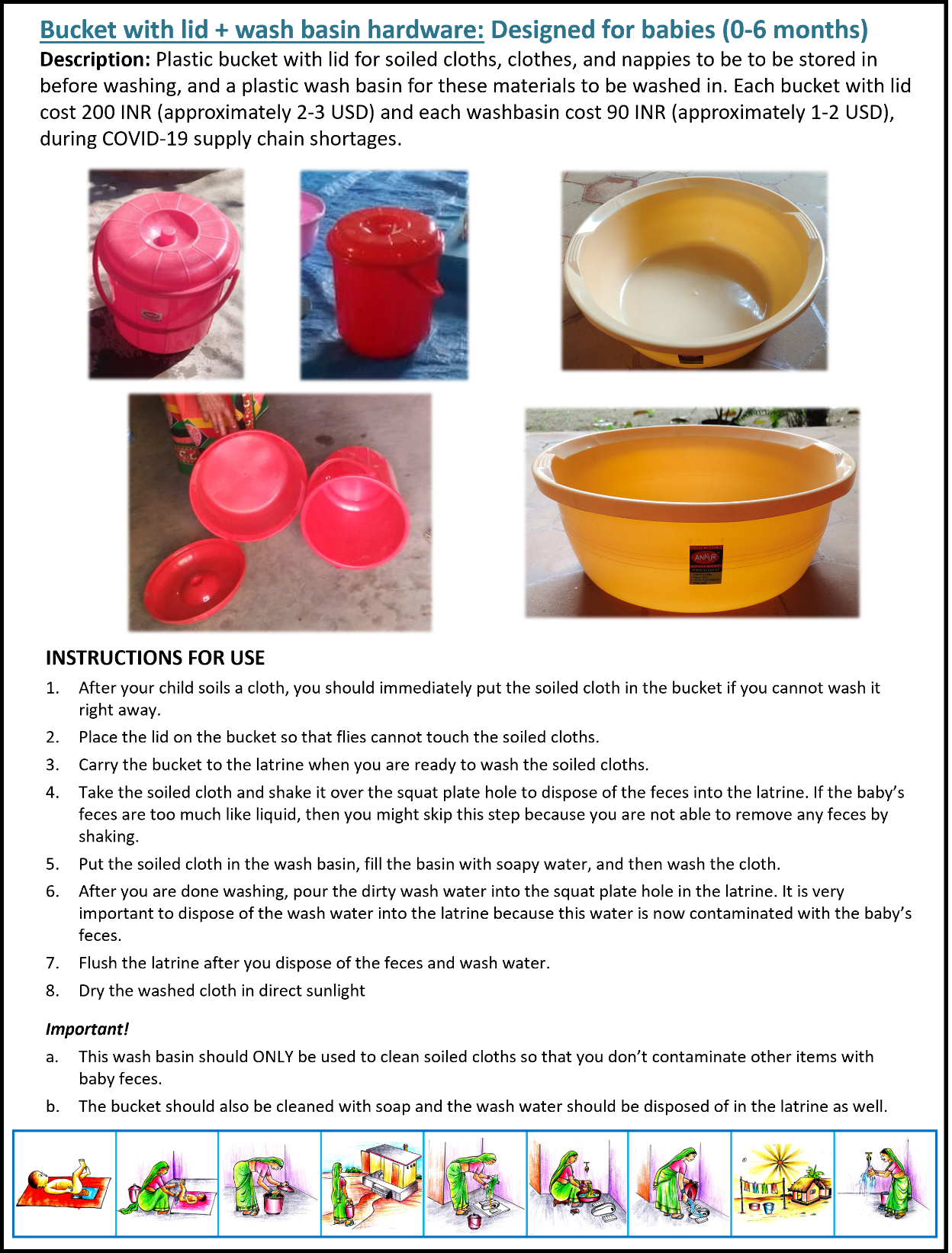


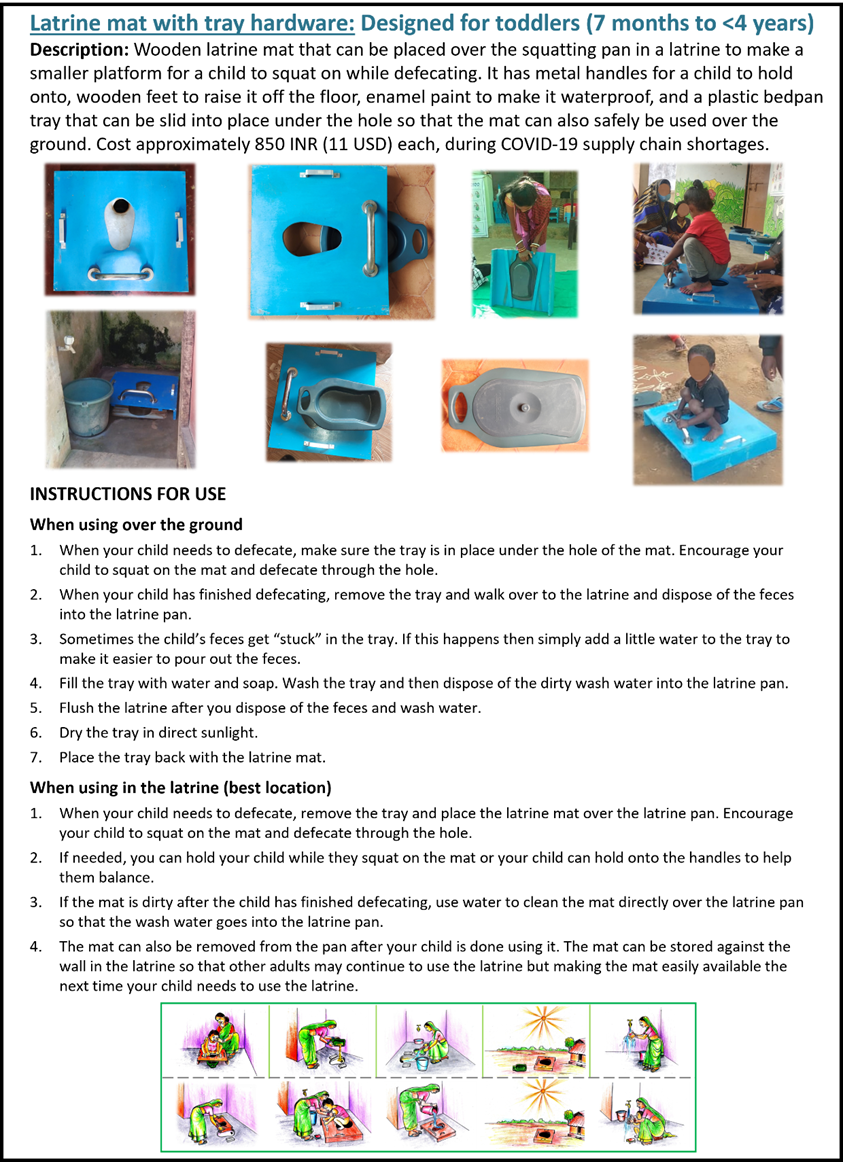

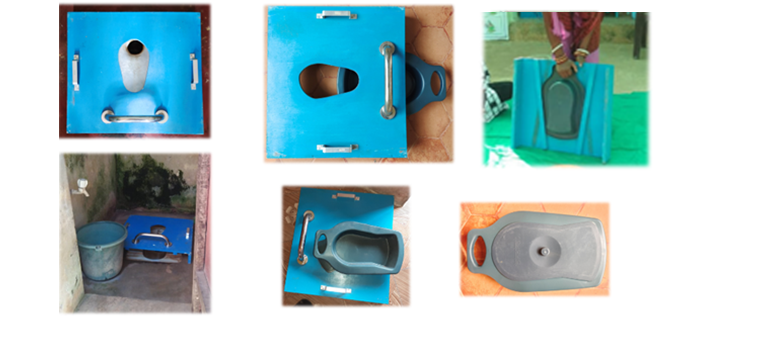

Supplement: Supplementary file 1 — Supplementary Material 1 [file 12889_2024_19272_MOESM1_ESM.docx]
