# Supplementary material for: Effect of a behavior change and hardware intervention on safe child feces management practices in rural Odisha, India: a cluster-randomized controlled trial
Source: BMC Public Health. 2024 Aug 27;24:2327. doi: 10.1186/s12889-024-19272-5 (PMC11351010; doi:10.1186/s12889-024-19272-5)
Supplement: Supplementary file 2 — Supplementary Material 2 [file 12889_2024_19272_MOESM2_ESM.docx]

**SUPPLEMENTAL File 2: Research Reflexivity Statement**

**Title:** Effect of a behavior change and hardware intervention on safe child feces management practices in rural Odisha, India: a cluster-randomized controlled trial

1. **How does this study address local research and policy priorities?**

This study reports on an intervention to improve child faeces management in a setting in rural Odisha, India where unsafe disposal of child faeces is highly prevalent and a likely contributor to the high levels of intestinal infection, diarrhoeal diseases and stunting. Gram Vikas, an Odisha-based NGO that served as the implementing partner, specifically connected with Emory University (study team) to work on safe child faeces disposal. Gram Vikas selected this health topic as a priority for the NGO and sought to develop a behaviour change program that could be incorporated into their broader community development programming.

1. **How were local researchers involved in study design?**

Local investigator Alokananda Bisoyi assisted in the design of survey tools and in their translation into Oriya. He hired, trained, and managed data collection teams and conceived of the ‘geo-demo’ stratification variable. Together with other authors, Mr. Bisoyi contributed to data interpretation, commented on drafts of the paper, approved the final version and agreed to the decision to submit for publication.

1. **How has funding been used to support the local research team?**

Mr. Biosoyi hired, trained and managed a team of 24 local staff involved in securing informed consent and in the collection of the study data. Most of these staff had little experience with field research at the commencement of the study, but were able to gain the skills and experience necessary to position themselves for further work in this area. Meantime, Mr. Biosoyi has commenced a masters in public health degree at JHU-affiliated IIHMR funded by the researchers.

1. **How are research staff who conducted data collection acknowledged?**

See the acknowledgement section of the manuscript where data collection team members are listed by name. We also acknowledge the implementing partner Gram Vikas team who were intentionally separated from the research team and are therefore not identified as authors.

1. **Do all members of the research partnership have access to study data?**

All members of the partnership have access to data as do any other investigators who may request the same.

1. **How was data used to develop analytical skills within the partnership?**

Co-author Biosoyi is a social scientist and participated in decisions about the analysis and reporting of the data. Data collectors were provided information on how data would be cleaned, analysed and reported.

1. **How have research partners collaborated in interpreting study data?**

As described in the manuscript, Gram Vikas leadership and staff were involved in intervention design and delivery approach. Stakeholder workshops — with representatives from government, community-based development organizations, and international NGOs (e.g. WaterAid, UNICEF) — were held at the start and end of the study to elicit input on intervention design, provide feedback on trial results, and promote dissemination. Data collectors participated in reviews of the completeness and quality of data both at baseline and endline. They also attended gatherings at which the results and reasons were discussed and where ideas were exchanged on how the intervention could be improved.

1. **How were research partners supported to develop writing skills?**

The entire authorship group participated in the development of the manuscript reporting the results. Field staff advanced their skills in the development and interpretation of survey tools and, especially, in collecting data on tablet-based devices using open source software. These are important data collection tools that will help staff obtain future positions in research.

1. **How will research products be shared to address local needs?**

The results were communicated to our implementing partner, Gram Vikas, in a series of meetings and workshops. These were also open to Odisha-based government officials, implementers and donors. The results were also reported to representatives of the funder, The Bill & Melinda Gates Foundation, which has a major presence in the country and which has influence with state and national policymakers.

1. **How is the leadership, contribution and ownership of this work by LMIC researchers recognised within the authorship?**

Co-author Biosoyi is an author of the paper. The paper includes specific information on his role and contributions.

1. **How have early career researchers across the partnership been included within the authorship team?**

Co-author Sclar led the research as part of her doctoral research at the University of Zurich. Co-author Bauza participated in the study as part of her post-doctoral fellowship at Emory University. Co-author Majorin participated in the study in her role as a junior researcher at the London School of Hygiene & Tropical Medicine.

1. **How has gender balance been addressed within the authorship?**

Three authors are women (Sclar, Bauza, Majorin) and three are men (Biosoyi, Mosler and Clasen).

1. **How has the project contributed to training of LMIC researchers?**

Most of the data collection staff had little experience with field research at the commencement of the study. The data collection teams received training in data collection techniques, including data entry on tablet-based devices using open source software. They also received training in data quality control and overall design and execution of field based environmental health research. These are highly transferable skills.

In addition, this study greatly contributed to co-author Bisoyi’s research training. This was Mr. Bisoyi’s first trial study and first time in the role of a local research manager. As such, throughout the course of the study and with support from fellow research team members, Mr. Biosoyi developed strong data collection skills, learned qualitative analysis, gained exposure to behaviour change theory and implementation science, and more. Mr. Bisoyi has now commenced a masters in public health degree at JHU-affiliated IIHMR.

1. **How has the project contributed to improvements in local infrastructure?**

This project was not intended to improve local infrastructure. Rather, it was to design, implement and rigorously assess a scalable intervention to improve health in rural setting through the safe disposal of child faeces. As we demonstrated the effectiveness and scalability of the intervention, we are hopeful that it will be implemented broadly.

1. **What safeguarding procedures were used to protect local study participants and researchers?**

The study protocol was reviewed and approved by the Institutional Review Board (IRB) of Emory University (IRB00115339) in Georgia, USA and the Independent Ethics Committee at Xavier University Bhubaneswar (220519) in Odisha, India. All participants provided verbal informed consent prior to their engagement in each trial survey. Researchers were protected by receiving extensive training, by being closely supervised, and by working in teams and having access to phones at all times in the field.
